# Differential sequences of exosomal NANOG DNA as a potential diagnostic cancer marker

**DOI:** 10.1371/journal.pone.0197782

**Published:** 2018-05-22

**Authors:** Manjusha Vaidya, Michael Bacchus, Kiminobu Sugaya

**Affiliations:** Burnett School of Biomedical Sciences, College of Medicine, University of Central Florida, Orlando, Florida, United States of America; University of South Alabama Mitchell Cancer Institute, UNITED STATES

## Abstract

NANOG has been demonstrated to play an essential role in the maintenance of embryonic stem cells, and its pseudogene, NANOGP8, is suggested to promote the cancer stem cell phenotype. As the roles of these genes are intimately involved with glioblastoma multiforme progression and exosomes are critical in intercellular communication, we conducted a detailed analysis of the association of the NANOG gene family with exosomes to identify diagnostic markers for cancer. Exosomes were precipitated from conditioned culture media from various cell lines, and NANOG gene fragments were directly amplified without DNA isolation using multiple primer sets. The use of the enzymes AlwNI and SmaI with restriction fragment length polymorphism analysis functioned to distinguish NANOGP8 from other NANOG family members. Collectively, results suggest that the NANOG DNA associated with exosomes is not full length and that mixed populations of the NANOG gene family exist. Furthermore, sequence analysis of exosomal DNA amplified with a NANOGP8 specific primer set frequently showed an insertion of a 22 bp sequence into the 3’ UTR. The occurrence rate of this insertion was significantly higher in exosomal DNA clones from cancer cells as compared to normal cells. We have detected mixed populations of NANOG DNA associated with exosomes and have identified preferential modulations in the sequences from cancer samples. Our findings, coupled with the properties of exosomes, may allow for the detection of traditionally inaccessible cancers (i.e. GBM) through minimally invasive techniques. Further analysis of exosomal DNA sequences of NANOG and other embryonic stemness genes (OCT3/4, SOX2, etc.) may establish a robust collection of exosome based diagnostic markers, and further elucidate the mechanisms of cancer formation, progression, and metastasis.

## Introduction

Exosomes are extracellular microvesicles (30–100 nm) released by almost all types of cells upon fusion of its multi-vesicular body with the plasma membrane. Known for their role in cell to cell communication, exosomes have demonstrated an ability to unload their contents and contribute to the transformation of normal and stem cells to cancerous states [1, 2]. Previous studies have suggested that RNAs associated with glioblastoma microvesicles may provide diagnostic biomarkers for cancer [3] and Thakur et al., have suggested that the double stranded DNA in cancerous exosomes could also serve as a marker [4]. In our current study, we have found DNA associated with exosomes produced not only by cancerous cells but also by normal cells. Because of this, we sought to investigate modifications of exosomal DNA that could function as diagnostic markers for cancer.

Glioblastoma multiforme (GBM) is the most common brain cancer, with most patients having an average life expectancy of less than two years following diagnosis. Researchers have previously reported increased methylation of the promoter for O^6^-methylguanine-DNA methyltransferase and mutations in the gene isocitrate dehydrogenase 1 as possible biomarkers in GBM tissue [5, 6]. However, recent studies have demonstrated that these markers are positively correlated with increased survival of the patient and suggest these targets better serve as prognostic rather than diagnostic markers [7, 8]. Because of this, the identification of accessible and accurate biomarkers to diagnose GBM in its early stages is still needed.

NANOG is a DNA binding homeobox transcription factor involved in maintaining the stemness of embryonic stem cells. Our group has previously reported increased expression levels of NANOG in GBM cancer stem cells, and have postulated that NANOG may be an ideal marker for the identification of cancer stem cells [9]. The NANOG family is comprised of the original NANOG gene and ten associated pseudogenes (P1-P10). Recently, researchers have shown increased expression levels of NANOGP8 in glioblastoma cancer stem cells and have suggested that NANOGP8 may participate in the reprogramming of normal cells into cancerous states [10, 11]. Furthermore, various studies have also reported increased expression levels of NANOG in cancer stem cells in other types of cancers, such as breast and lung cancers, supporting that NANOG and NANOGP8 are established cancer markers [12, 13, 14, 15]. Based on these findings, we analyzed the NANOG DNA sequence associated exosomes derived from a variety of cell sources to identify characteristics that could serve as diagnostic markers of cancer.

## Methods and materials

### Cell culture

Human Neuronal Stem Cells or F-HNSC (procured as Fetal-derived human neural progenitor cells from Lonza) and Glioblastoma Multiforme cells (GBM) were grown in suspension cultures. Primary GBM was prepared by dissociation of human brain tumor patient specimens in accordance with a protocol approved by Florida Hospital Institutional Review Board. The subjects were given informed consent and HIPPA regulations were strictly followed. For proliferation, the cells were cultured in HNSC media containing Heparin 5000 U (0.5 U / mL), EGF—20 ng/mL, bFGF—20 ng / mL and 2% B27 stock mixed in DMEM/F12. To differentiate these cells, the cells were cultured in NT2 (NTERA-2 human embryonal carcinoma cell line) media containing DMEM-F12 supplemented with 10% exosome-depleted FBS. Following the manufacturer’s protocol, the cancer stem cells were separated from proliferating GBM cells using CD133 conjugated magnetic beads (Miltenyi Biotec, CD133 microbeads, human, Mat. No. 120-000-312). HEK293 cells (ATCC) were cultured in DMEM containing 10% exosome-depleted FBS, L-glutamine, and 100x nonessential amino acids. Primary umbilical cord blood derived AC133 positive endothelial progenitor cells (AC133) were cultured in hematopoietic progenitor media containing 500 μL of the cytokine FLT3 (fms like tyrosine kinase 3), 500 μL uL of the cytokine SCF (stem cell factor) and 100 μL of the cytokine TPO (thrombopoietin). All types of media were supplemented with penicillin and streptomycin (100 U / mL of each). The following spent media was provided by Dr. Annette Khaled Lab: Conditioned BMEM media for normal bronchial epithelial cells (CRL9609), conditioned BMEM media for normal breast epithelial cells (MCF-10A,), conditioned HITES media for small cell lung cancer (CRL5903), and conditioned DMEM media for triple negative breast cancer (MDA-MB-231) cells.

### Exosome isolation

The spent media was centrifuged at 10000xg for 30 minutes to remove cell debris. Exosomes were isolated from conditioned culture media using a modified PEG-NaCl precipitation method [16]. In brief, 10 mL of supernatant was used to precipitate exosomes through the addition of 5 mL of 20% PEG and 200 μL of 7.5 M NaCl and subsequent overnight incubation at 4° C. The following day, the supernatant was centrifuged at 10000xg for 60 minutes and the exosome pellet was re-suspended in 1x PBS (pH 7.4, sans Calcium and Magnesium). Using CD63 conjugated magnetic beads [Invitrogen by Thermo Fisher Scientific Exosome—Human CD63 Isolation/Detection (from cell culture media), Ref- 10606D], the exosomes were further purified following the manufacturer’s protocol.

### PCR settings and electrophoresis

The exosomes were used directly in place of template without DNA extraction. Using High-Performance GoTaq^®^ G2 DNA Polymerase (Promega), the PCR reactions were set up as follows: 94°C -5 minutes, (denaturation: 94°C—30 seconds, annealing: 55°C—30 seconds, Extension: 72°C– 2 minutes)x30, 72°C -10 minutes. The PCR products were run on 1.5% Agarose gel in 1X TAE buffer.

### Restriction enzyme digestion of the PCR products

The DNA with extracted with the QIAquick Gel Extraction Kit (Qiagen), using the manufacturer’s protocol. 1 μg of amplicon DNA was digested with restriction enzymes AlwNI (also known as CaiI), and *Sma*I (both ThermoFisher, Fast Digest enzymes) with Fast Digest buffer, at 37° C for five minutes and run on 3% Agarose gel in 1X TAE buffer.

### Cloning of PCR products in pCR4TOPO-TA vector

Following the manufacturer’s protocol, the PCR products were ligated with the vector, transformed into chemically competent E. coli (Stbl3) cells, and selected on LB with ampicillin (100 μg/mL). Upon overnight incubation at 37°, the colonies were picked, grown in LB with Ampicillin, and the DNA was extracted using QIAprep Spin Miniprep Kit. The clones were digested with Fast Digest (FD) restriction enzyme EcoRI for five minutes at 37° C and run on 1.5% Agarose gel in TAE buffer.

### Exosome sample preparation for imaging

To confirm the integrity of the purified exosomes’ structure, we transfected HEK293 cells with XPack MSCV-XP-RFP-EF1 α-Puro vectors (SBI) to express red fluorescent protein (RFP) on the inner surface of the exosomal membrane. Using transfection reagent Lipofectamine^®^ 2000, ~80% confluent HEK293 cells were transfected with 10 μg of plasmid DNA following the manufacturer’s protocol. Within 24 hours, the HEK293 cells were observed for RFP and exosomes were collected according to the protocol previously described. The green fluorescent, lipophilic carbocyanine DiO dye (ThermoFisher Scientific) was reconstituted using the manufacturer’s protocol. Exosomes were incubated at room temperature for one hour with the stain. Using exosome spin column (Invitrogen by Life Technologies, exosome spin columns, mw 3000, Ref. 4484449), excess unbound dye was removed.

### Confocal imaging

To prepare the samples for confocal microscopy, glass slides were coated with poly L-Ornithine. 50 μL of the DiO stained, RFP packed HEK293 exosome suspension was smeared. After letting the slide dry for five minutes, a cover slip was placed and sealed with transparent acetone. The stained exosomes were imaged using the Zeiss 710 with the Zeiss AxioObserver microscope and the objective plan apochromat 63x / 1.40x Oil DIC M27. Green fluorescence was detected at an excitation wavelength setting of 488 nm and emission wavelength setting of 542 nm. Red fluorescence was detected at an excitation wavelength setting of 543 nm and emission wavelength setting of 675 nm.

### Statistics

To analyze the occurrence rate of the 22 base pair insertion into the 3’ UTR of NANOGP8, one-way ANOVA was performed. To identify the significance of this variation between cancer cell derived exosomes and non-cancer cell derived exosomes, one-way ANOVA was followed by post hoc analysis via Student-Newman-Keuls tests.

## Results

**Fig 1:**
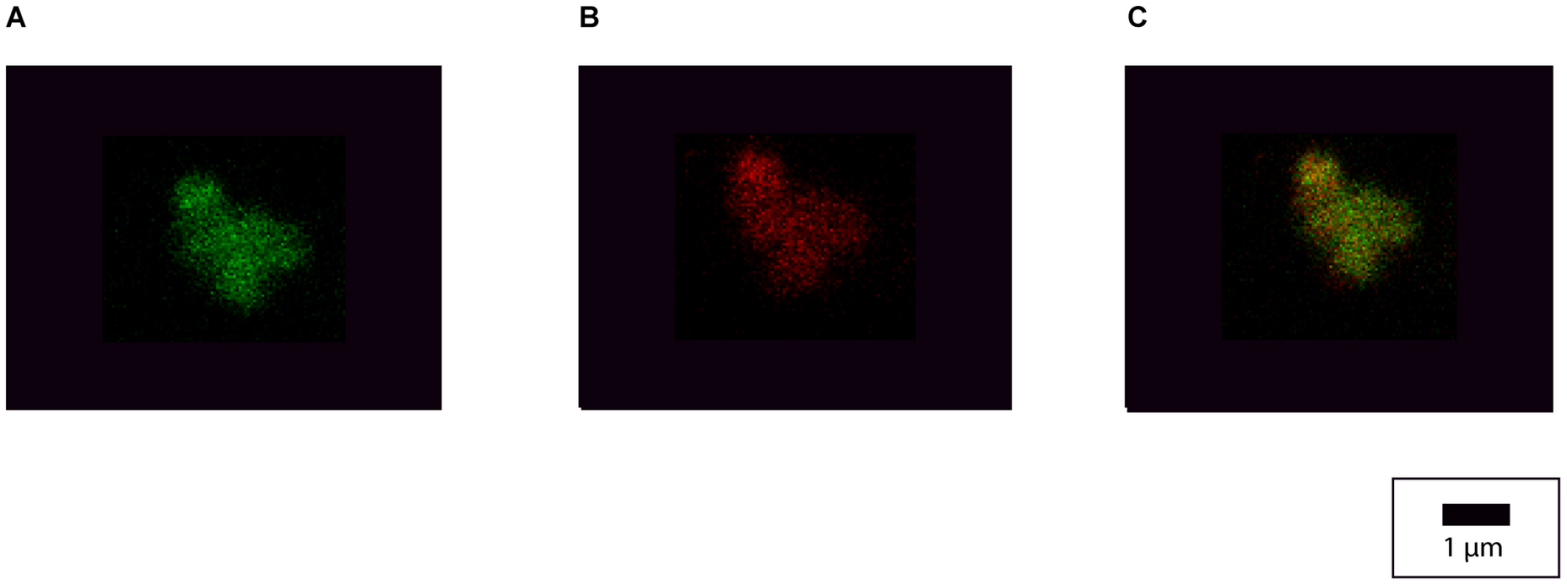
Confocal microscopy of HEK 293 exosome clusters. Confocal microscopy images of HEK 293 exosome clusters, demonstrating the preserved integrity of exosome structure of the samples used within this study. (A) Green fluorescence from lipophilic stain DiO. (B) Red fluorescence from packed RFP (red fluorescent protein). (C) Co-localization of both DiO staining and RFP.

Colorization of RFP on the inside and DiO green fluorescence on the outside of the exosomal membranes was observed by confocal microscopy, confirming the presence of intact exosomes in our samples preparation (Fig 1A, 1B and 1C).

**Fig 2:**
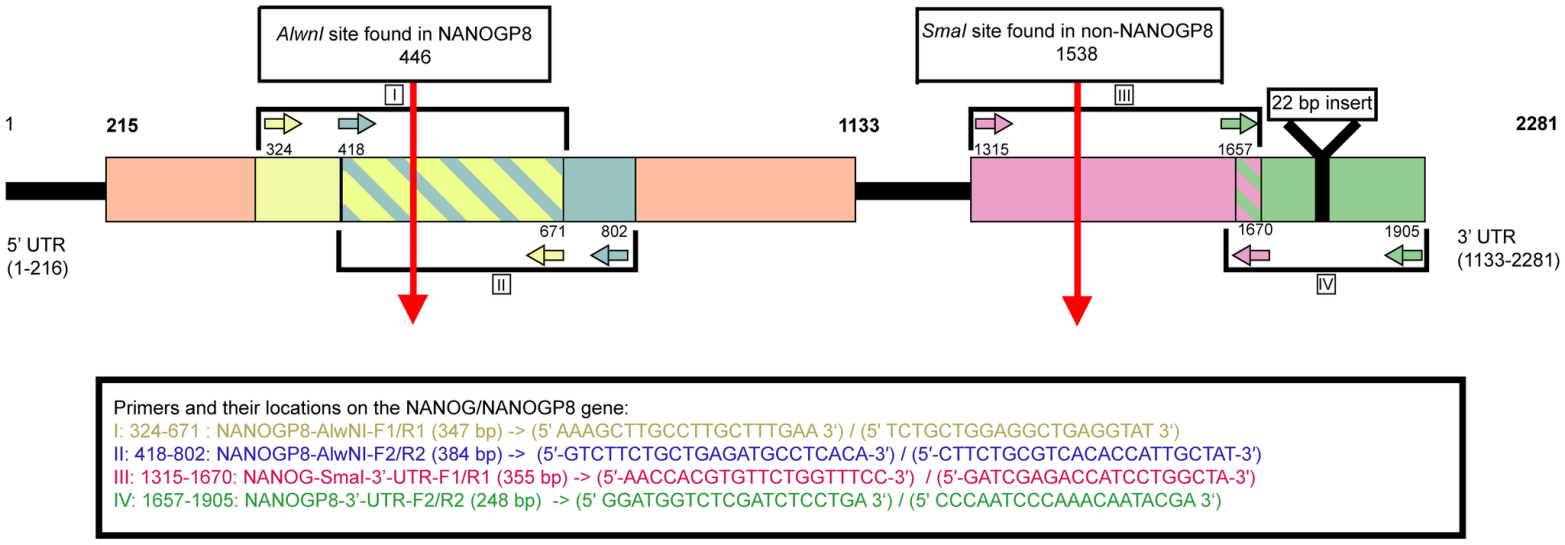
NANOGP8 gene and primer map. Primer sequences (I-IV) and locations of their PCR products in NANOGP8 gene (GenBank Accession # NG_004093.3). Locations of the AlwNI and SmaI restriction enzyme cutting sites are indicated by red arrows. PCR products with primer pair IV frequently had an insertion of a 22 bp fragment at position 1773 in the 3’ UTR of NANOGP8 mRNA (GenBank Accession # NM_001355281.1).

**Fig 3:**
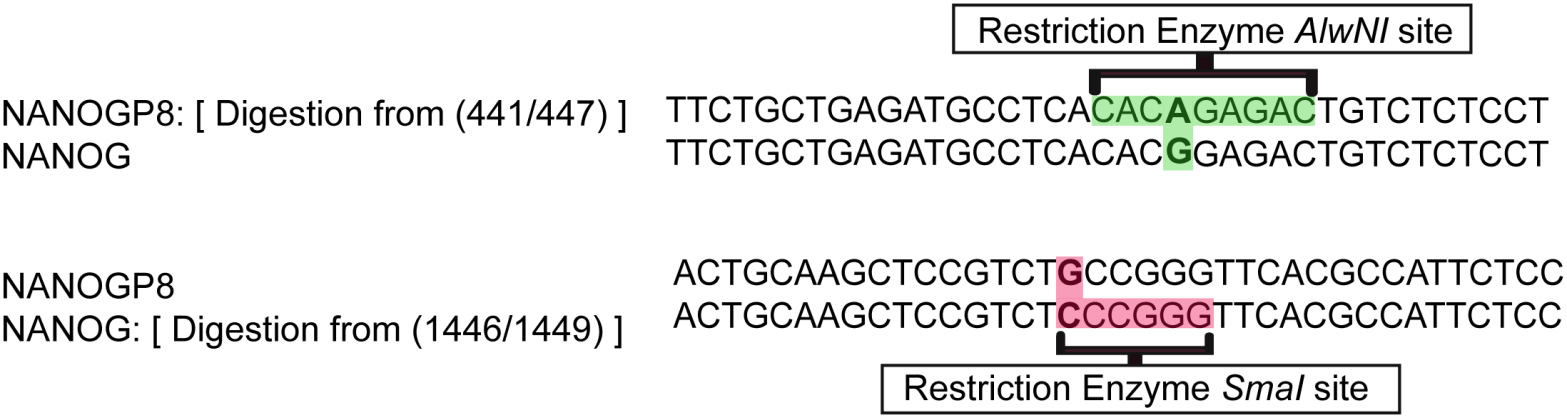
Restriction enzyme sequences. Comparison of restriction enzyme cutting sites between NANOGP8 and other NANOG family members. AlwNI site is present and SmaI site is absent in NANOGP8. A single nucleotide at 442 from A to G in NANOG P8 (when compared to other NANOG genes) produces an AlwNI site, while a single nucleotide substitution at 1535 from C to G removes a SmaI site.

The gene fragments associated with PEG-NaCl precipitated exosomes were directly amplified by PCR without DNA isolation using primer sets described in (Fig 2). The identities of the PCR products were confirmed through restriction fragment length polymorphisms (RFLP) analysis by the identification of AlwNI or SmaI digestion (Fig 3). Primer sets I and II amplified regions containing the AlwNI site unique for NANOGP8 at nucleotide position 446 (GenBank Accession: 388112). The difference in binding positions between primer sets I and II is a 94 bp shift in the forward primer and 131 bp shift in the reverse primer. Primer set III amplified a region containing the SmaI restriction enzyme site unique for NANOG at position 1457 (GenBank Accession: 79923). Primer set IV is specific for a region in the 3’ UTR of NANOGP8 [17, 18]. A summary of the results, including the presence or absence of NANOGP8 based on cell source and primer set, is reflected in Table 1.

**Table 1:** Summary of PCR product analyses.

	NSC	NSC	GBM	GBM	GBM	GBM	HEK 293	AC133	CRL9609	CRL5903	MCF-10A	MDA-MB-231
	Proliferating	Differentiated	Proliferating	Differentiated	CD133-	CD133+						
**Primer Set I**	+ / -	+ / -	+ / -	+ / -	NA	+ / -	NA	+ / -	NA	NA	NA	+ / -
	D	D	D	D	R	D	R	D	R	R	R	D
**Primer Set II**	+ / -	+ / -	+ / -	+ / -	+ / -	+ / -	+ / -	+ / -	+ / -	+ / -	+ / -	+ / -
	S	S	S	D	D	D	D	D	D	D	D	D
**Primer Set III**	+ / -	+ / -	+ / -	+ / -	+ / -	+ / -	- / -	+ / -	+ / -	+ / -	- / -	+ / -
	S	D	S	D	D	D	D	D	D	D	D	D
**Primer Set IV**	25%	33%	75%	75%	40%	60%	100%	100%	60%	93.33%	60%	93.33%
	S	S	S	S	S	S	S	S	S	S	S	S

+ / - represents a mixed population of NANOGP8 and other NANOG genes. - / - represents a complete absence of NANOGP8. NA: No PCR product was created with the given primer set. S: PCR product underwent sequence analysis. D: PCR product underwent RFLP analysis. R: PCR product was re-amplified by PCR to confirm the absence of the target sequence in the sample. Percentages in row 4 indicate percentage of clones containing the 22 bp insertion.

**Fig 4:**
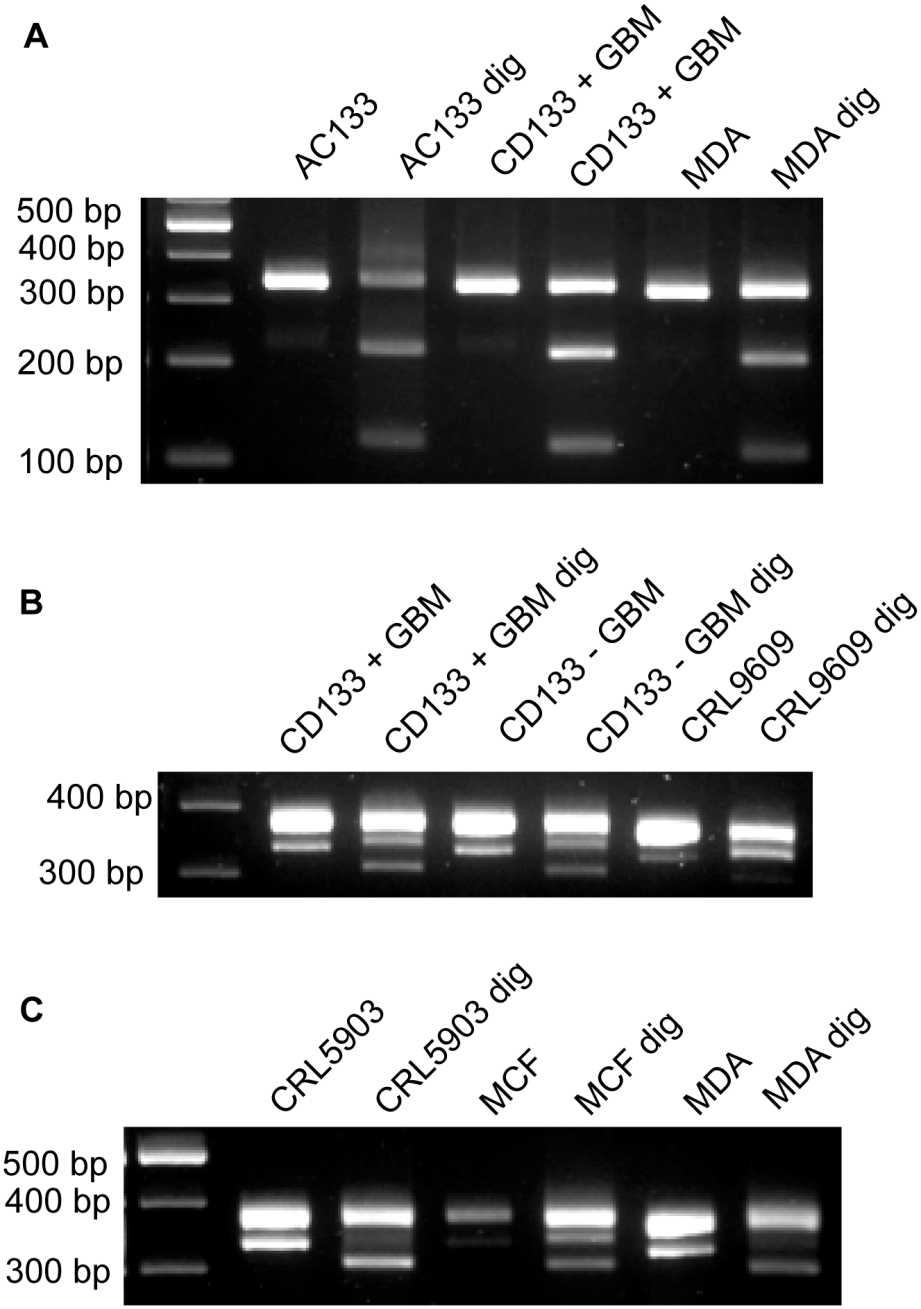
Typical gel images of exosomal DNA fragments amplified and digested (dig) with AlwNI. (A): PCR products from Primer set I. (B, C): PCR products from Primer set II.

We observed both undigested (347 bp) and digested (225 bp and 122 bp) products with electrophoresis after extensive AlwNI treatment of the PCR products generated by primer set I in all the samples analyzed (Fig 4A). Additionally, AlwNI digestion of PCR products amplified by primer set II showed undigested (384 bp) and digested (356 bp) products, but the presence of a small 28 bp fragment was not detectable in all exosome samples (Fig 4B and 4C). In addition to the gel electrophoresis, the sequence of the PCR products was analyzed by a M13 sequencing primer after cloning the PCR fragments into pCR4TOPO-TA vectors. For a given cell type, sequence analysis identified an AlwNI cutting site in some clones but not all indicating that a mixed population of NANOGP8 and other NANOG family members was present in the DNA fragments associated with exosomes.

While primer set II successfully yielded 347 bp PCR products with exosomes isolated from all cell types, primer set I failed to generate PCR products from five sources (HEK293, CD133 negative GBM, CRL9609, CRL5903, and MCF-10A). The absence of a PCR product after such a shift may derive from exosomal NANOG DNA lacking a region containing the 94 base pair sequence. Other possible causes include a lack of a primer binding site for the forward and/or reverse primers, as well as nucleotide polymorphisms at the primer binding site for these five types of exosomes. The cell source specific absences of exosomal NANOGP8 regions could serve as a potential marker, and warrants further study.

Furthermore, attempts to amplify the full length of NANOG (218–2093 bp) by PCR [19] failed to obtain any product with exosomal DNA isolated from NSCs. This contrasts to when we used cellular DNA of NSCs as the template, which successfully amplified the 1875 bp full length NANOG (results not shown). These results suggest that the DNA associated with exosomes may not contain the full length of NANOG but with regions absent.

**Fig 5:**
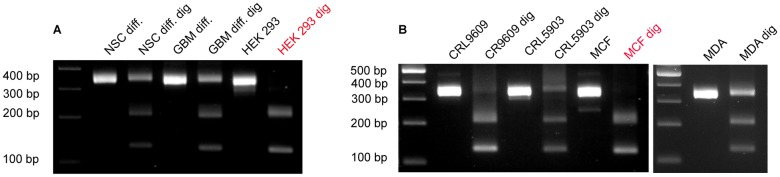
Typical gel images of exosomal DNA fragments amplified and digested with SmaI. Red letters indicate a complete digestion by SmaI due to the absence of NANOGP8 in these samples.

The majority of RFLP analyses of products amplified using primer set III and digested with SmaI showed partial digestion, indicating mixed populations of NANOGP8 and other NANOG family genes. Exosome samples isolated from HEK293 and MCF-10A, however, showed a complete digestion with SmaI through production of 213 bp and 142 bp fragments (Fig 5A and 5B, red font), suggesting that the DNA fragments associated with these exosomes contain only NANOG (GenBank Accession: 79923) from position 1315–1670.

**Fig 6:**
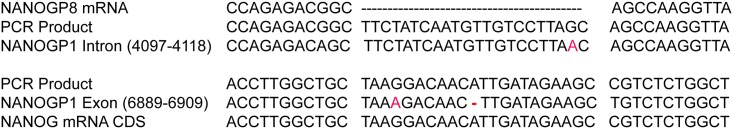
NANOGP8 insertion sequence analysis. The insert and the surrounding area had high homology to the second intron of NANOGP1 (insertion point 4097, GenBank Accession # NG_006522.3), the fourth exon of NANOGP1 (insertion point 6889, GenBank Accession # NG_006522.3), and NANOG mRNA cds (insertion point 1683, GenBank Accession # AB093576.1).

Sequence analysis of pCR4TOPO-TA vector-cloned PCR products from all samples amplified with primer set IV [20], which is specific to NANOGP8 (GenBank Accession: 388112), showed an insertion of a 22 bp sequence at position 1773. The 22 bp sequence has not been reported in NANOGP8 mRNA (GenBank Accession: NM_001355281.1) nor NANOGP8 genomic DNA (GenBank Accession: NC_000012.12), and thus can be considered an insertion. Although the 22 bp insert is reported in the coding sequence region (cds) of NANOG (GenBank Accession: AB093576.1) and the second intron (4097–4118) as well as the fourth exon (6809–6909) of NANOGP1 (GenBank Accession: 404635), the PCR fragments cannot belong to these genes as primer set IV is NANOGP8 specific. The adjacent sequences, up to 18 bp on either side of the insert, of the PCR products have 94.4% to 100% homology to the corresponding regions of NANOG cds, NANOGP1 intron 2, and NANOGP1 exon 4. This suggests that the insertion always occurs within a specific sequence for these genes. Furthermore, we observed nucleotide polymorphisms in the 22 bp insert between the PCR products and corresponding sequence of the NANOGP1 exon or intron (Fig 6).

**Fig 7:**
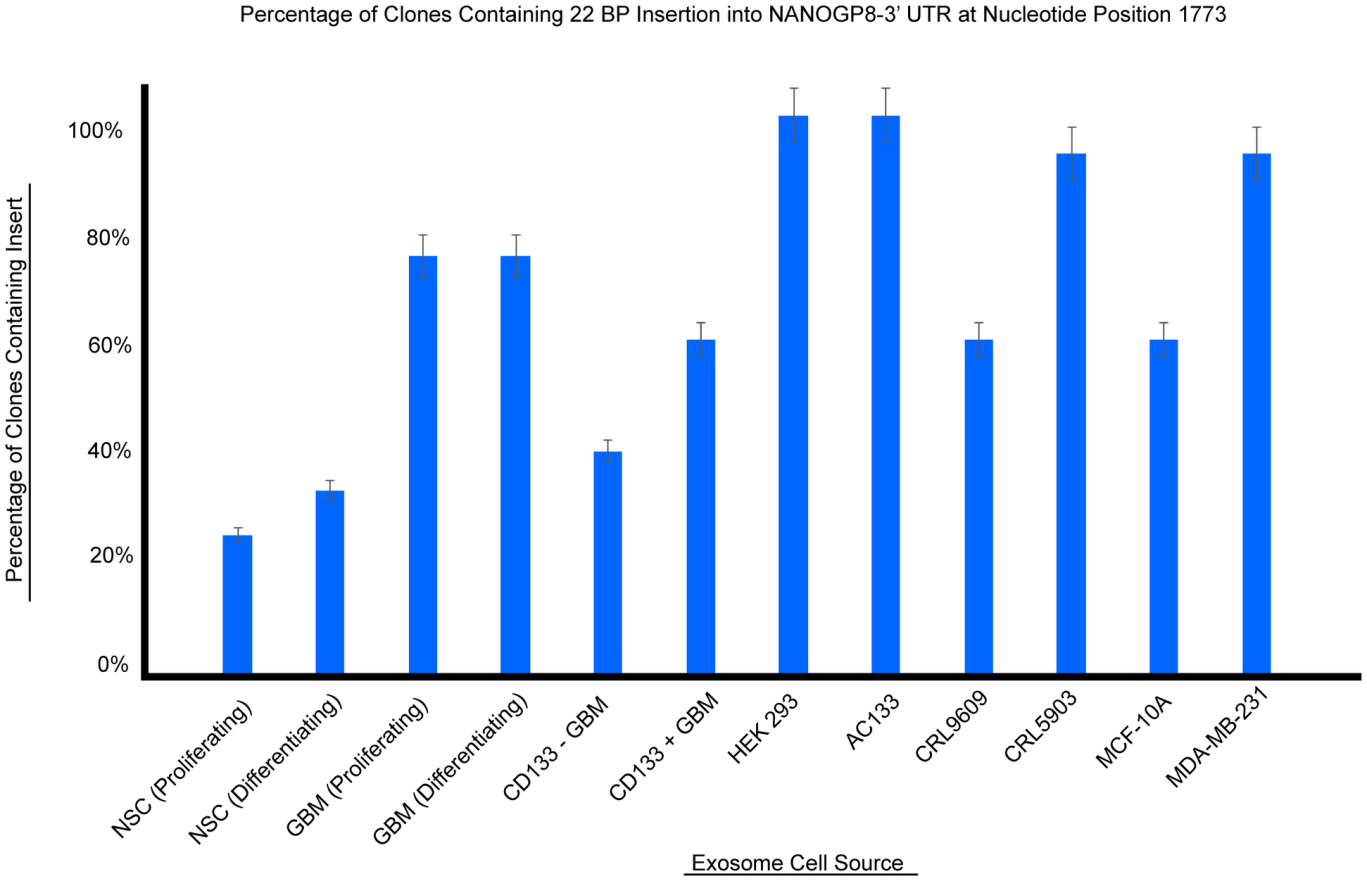
22 BP insertion occurrence. Percentage of clones within a cell source containing the 22 bp insert into NANOGP8 3’UTR of exosomal DNA. Specific values are given in Table 1, primer set IV.

**Fig 8:**
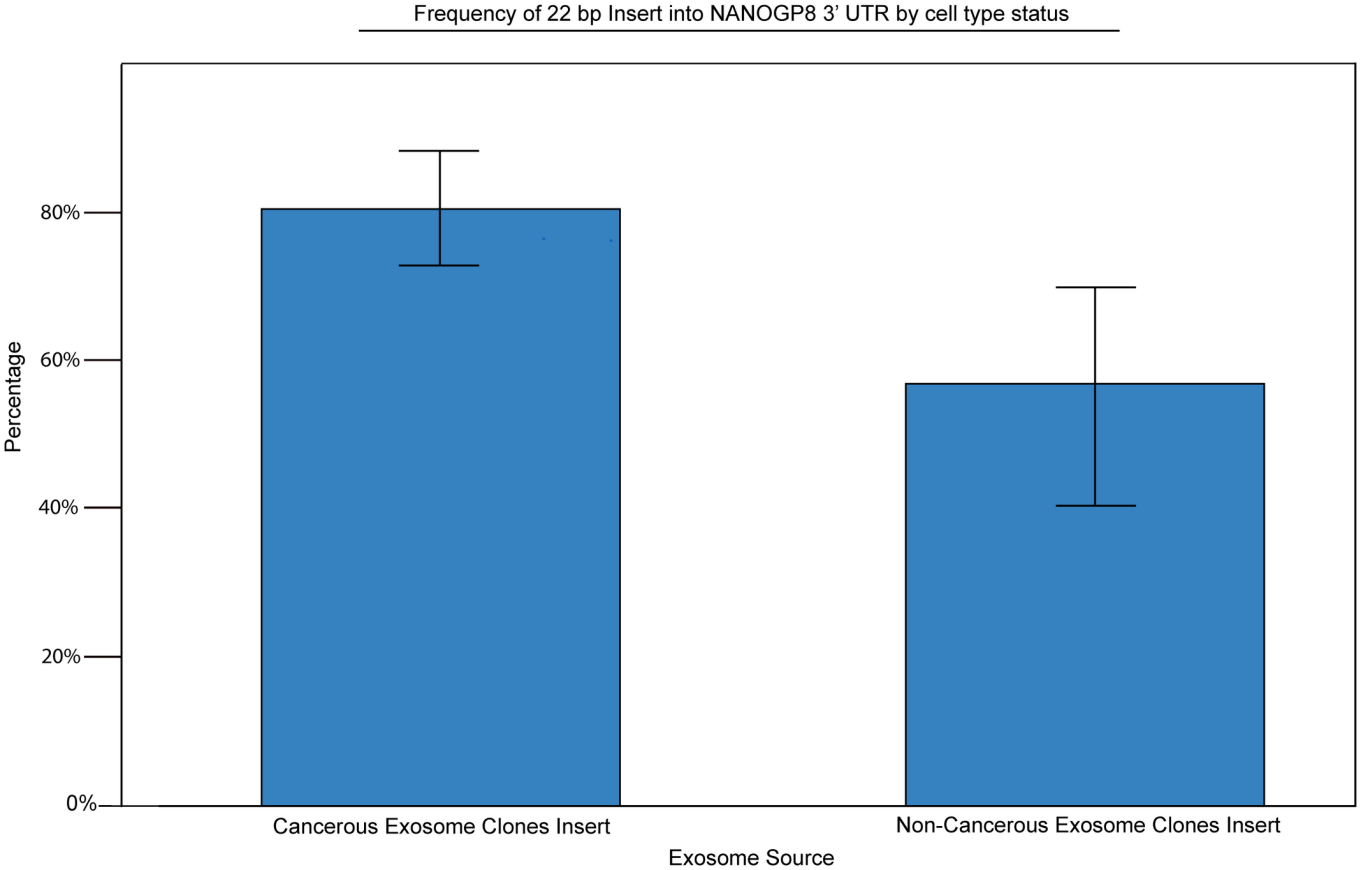
22 BP insertion comparison based on cell source status. Analysis demonstrated a comparative result of $\bar{\text{x}}=0.8036$ with σ = ± 0.4009 for cancerous cell derived exosomes, versus $\bar{\text{x}}=0.5768$ with σ = ± 0.5038 for noncancerous cell derived exosomes (with 1 = insertion present and 0 = insertion absent.) The usage of a confidence interval at C = 0.85 produces intervals of (0.7254, 0.8818) and (0.4302, 0.7237) for cancerous and noncancerous derived exosomes, respectively.

The percentage of clones positive for the 22 bp insert varies depending upon the cell source of the exosomes (Fig 7). To compare the occurrence of the insertion between exosomes derived from cancerous and non-cancerous cells, one-way ANOVA followed by post hoc analysis via Student-Newman-Keuls tests was used. At α = 0.15, a F value of 4.07 was calculated in addition to p = 0.05 and Pr > F = 0.0313. In addition to calculated confidence intervals, analysis of the distribution of the data and mean values for the insertion in cancerous versus noncancerous derived sources demonstrate that the mean values are significantly apart (Fig 8). These results indicate that exosomal DNA derived from cancerous cells have a significantly higher percentage of the insert when as compared to their normal counterparts. Although cell lines MCF 10A and CRL9609 were classified as noncancerous in this study, these cell lines may exhibit cancer-like properties as these are immortalized cell lines. Such cell lines may contain a higher rate of the insertion than normal primary cell lines. Furthermore, because this insertion occurs within the 3’ UTR of the NANOG gene family, it may play a role in modifying translation of the gene [21]. Similarly, the size of the insert (22 bp) may indicate mechanisms related to microRNA [22]. Further detailed studies are needed to identify the significance of the insert sequence and functions involving cancer initiation and growth.

## Discussion

The critical roles of NANOG and NANOGP8 in cancer progression leads the association of these genes with exosomes to be significant, and may allow for exosomal NANOG to function as a powerful diagnostic biomarker. Variations in NANOG/NANOGP8 gene sequences in exosomal DNA, including an insertion into the 3’ UTR and a complete absence of certain gene regions, present novel characteristics that warrant further study. Moreover, recent studies have shown that extracellular vesicles, including exosomes, are capable of crossing the blood brain barrier and therefore are detectable in the peripheral blood via minimally invasive techniques [23]. Thus, our finding of the existence of exosomal NANOG DNA allows for the potential to develop unique diagnostic tools for cancer in restricted locations (i.e. GBM). To increase the specificity of diagnoses, continued studies to examine other known stemness genes, such as OCT3/4 and SOX2, should be pursued. Further studies should also explore variances within modulated exosomal NANOG DNA among specific stages of cancer, to identify any prognostic implications. Because NANOG expression is increased in cancer stem cells and research has suggested that NANOGP8 may be involved in the reprogramming of normal cells to cancer cells, the identification of exosomal NANOG DNA fragments provides further insight on the mechanisms of cancer formation and metastasis.

## Supporting information

S1 Fig EcoRI digestion of PCR product clones. Each lane contains exosomal DNA PCR products derived from proliferating human neural stem cells. Lane 1 contains the DNA ladder (GeneRuler DNA Ladder Mix). Lanes 2–5 contain samples amplified using NANOG/P8-*SmaI*-3’-UTR-F2/R2 (Primer set III). Lanes 6–7 contain samples amplified with NANOG/P8-3’UTR-F2/R2 (Primer set IV). Lanes 2, 4, and 6 contain undigested samples (controls) and lanes 3, 5, and 7 contain samples digested with EcoRI. As the pCR4-TOPO-TA vector contains EcoRI sites flanking the cloned PCR product, digestion by EcoRI serves as a confirmation for a positive clone.
(PDF)

S2 Fig *SmaI* digestion of PCR product clones and analysis. A. Lane 1 contains the DNA ladder (GeneRuler DNA Ladder Mix). Lanes 2–3 contain exosomal DNA PCR products derived from proliferating human neural stem cells. Lanes 4–5 contain exosomal DNA PCR products derived from proliferating GBM cells. Lanes 2–5 contain samples amplified using NANOG/P8-*SmaI*-3’-UTR-F2/R2 (Primer set III). Lanes 2 and 4 contain undigested samples (controls) and lanes 3 and 5 contain samples digested with SmaI. As the pCR4-TOPO-TA vector lacks SmaI restriction enzyme sites, the digestion and linearization by SmaI is a confirmation for a positive clone. B. BLAST analysis of the clone of exosomal DNA PCR products derived from proliferating human neural stem cells seen in A. This pCR4-TOPO-TA vector clone contains the PCR fragment produced using NANOG/P8-*SmaI*-3’-UTR-F2/R2 (Primer set III). SmaI restriction enzyme site is indicated by a box. C. Analysis of the clone of exosomal DNA PCR products derived from proliferating GBM cells seen in A. This pCR4-TOPO-TA vector clone contains the PCR fragment produced using NANOG/P8-*SmaI*-3’-UTR-F2/R2 (Primer set III). SmaI site is indicated by a box.
(PDF)

S3 Fig PCR product of exosomal DNA and NANOG cds. Comparison of PCR product of exosomal DNA derived from small cell lung cancer CRL5903 with ‘NANOG Homo sapiens mRNA for homeobox transcription factor Nanog, complete cds’ (GenBank: AB093576.1). The exosomal DNA was amplified with NANOG/P8-3’UTR-F2/R2 (Primer set IV) and cloned into pCR4-TOPO-TA vector. The PCR product contains a sequence of 22 bp (indicated by a box) not reported in NANOG mRNA variants. This 22bp sequence is reported within NANOGP1 intron from positions 4097–4118 and within NANOGP1 exon from positions 6889–6909.
(PDF)

S4 Fig PCR product of exosomal DNA and genomic NANOG. Comparison of PCR product of exosomal DNA derived from small cell lung cancer CRL5903 with ‘NANOG genome sequence Homo sapiens chromosome 12, GRCh38.p7’ (NCBI Reference Sequence: NC_000012.12). The exosomal DNA was amplified with NANOG/P8-3’UTR-F2/R2 (Primer set IV) and cloned into pCR4-TOPO-TA vector. The PCR product contains a sequence of 22 bp (indicated by a box) not reported in NANOG genomic DNA. This 22bp sequence is reported within NANOGP1 intron from positions 4097–4118 and within NANOGP1 exon from positions 6889–6909.
(PDF)

S5 Fig PCR product of exosomal DNA and NANOG mRNA transcripts. Comparison of PCR product of exosomal DNA derived from small cell lung cancer CRL5903 with A. ‘Homo sapiens Nanog homeobox (NANOG), transcript variant 1, mRNA’ (NCBI Reference Sequence: NM_024865) and B. ‘Homo sapiens Nanog homeobox (NANOG), transcript variant 2, mRNA’ (NCBI Reference Sequence: NM_001297698.1). The exosomal DNA was amplified with NANOG/P8-3’UTR-F2/R2 (Primer set IV) and cloned into pCR4-TOPO-TA vector. The PCR products contain a sequence of 22 bp (indicated by a box) not reported in NANOG mRNA transcript variant 1 and 2. This 22bp sequence is reported within NANOGP1 intron from positions 4097–4118 and within NANOGP1 exon from positions 6889–6909.
(PDF)

S6 Fig PCR product of exosomal DNA and NANOGP8 cds. Comparison of PCR product of exosomal DNA derived from small cell lung cancer CRL5903 with ‘NANOGP8 cds Homo sapiens isolate NA07038*D2-1 NANOGP8 (NANOGP8) gene, complete cds’ (GenBank: JX104846.1). The exosomal DNA was amplified with NANOG/P8-3’UTR-F2/R2 (Primer set IV) and cloned into pCR4-TOPO-TA vector. The PCR product and NANOGP8 cds share 100 base pairs of homology.
(PDF)

S7 Fig PCR product of exosomal DNA and NANOGP8 on chromosome 15. Comparison of PCR product of exosomal DNA derived from small cell lung cancer CRL5903 with ‘Homo sapiens Nanog homeobox pseudogene 8 (NANOGP8) on chromosome 15’ (NCBI Reference Sequence: NG_004093.3). The exosomal DNA was amplified with NANOG/P8-3’UTR-F2/R2 (Primer set IV) and cloned into pCR4-TOPO-TA vector. The PCR product contains a sequence of 22 bp (indicated by a box) not reported in genomic NANOGP8. This 22bp sequence is reported within NANOGP1 intron from positions 4097–4118 and within NANOGP1 exon from positions 6889–6909.
(PDF)

S8 Fig PCR product of exosomal DNA and NANOGP8 mRNA. Comparison of PCR product of exosomal DNA derived from small cell lung cancer CRL5903 with ‘Homo sapiens Nanog homeobox retrogene P8 (NANOGP8), mRNA’ (NCBI Reference Sequence: NM_001355281.1). The exosomal DNA was amplified with NANOG/P8-3’UTR-F2/R2 (Primer set IV) and cloned into pCR4-TOPO-TA vector. The PCR product contains a sequence of 22 bp (indicated by a box) not reported in genomic NANOGP8 mRNA. This 22bp sequence is reported within NANOGP1 intron from positions 4097–4118 and within NANOGP1 exon from positions 6889–6909.
(PDF)
