# Supplementary figures and images for: Differential sequences of exosomal NANOG DNA as a potential diagnostic cancer marker
Source: PLoS One. 2018 May 22;13(5):e0197782. doi: 10.1371/journal.pone.0197782 (PMC5963750; doi:10.1371/journal.pone.0197782)

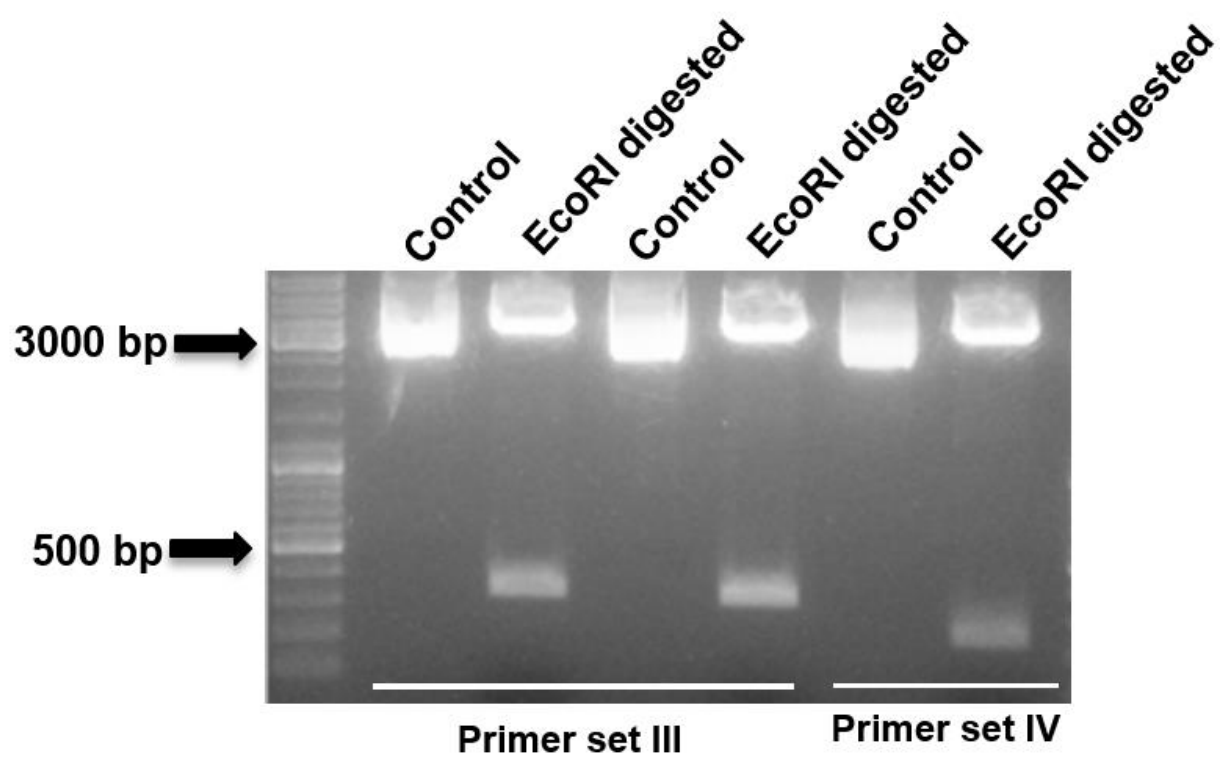

Supplement: S1 Fig — Each lane contains exosomal DNA PCR products derived from proliferating human neural stem cells. Lane 1 contains the DNA ladder (GeneRuler DNA Ladder Mix). Lanes 2–5 contain samples amplified using NANOG/P8-SmaI-3’-UTR-F2/R2 (Primer set III). Lanes 6–7 contain samples amplified with NANOG/P8-3’UTR-F2/R2 (Primer set IV). Lanes 2, 4, and 6 contain undigested samples (controls) and lanes 3, 5, and 7 contain samples digested with EcoRI. As the pCR4-TOPO-TA vector contains EcoRI sites flanking the cloned PCR product, digestion by EcoRI serves as a confirmation for a positive clone. (PDF) [file pone.0197782.s001.pdf]
