# Supplementary material for: Differential sequences of exosomal NANOG DNA as a potential diagnostic cancer marker
Source: PLoS One. 2018 May 22;13(5):e0197782. doi: 10.1371/journal.pone.0197782 (PMC5963750; doi:10.1371/journal.pone.0197782)
Supplement: S2 Fig — A. Lane 1 contains the DNA ladder (GeneRuler DNA Ladder Mix). Lanes 2–3 contain exosomal DNA PCR products derived from proliferating human neural stem cells. Lanes 4–5 contain exosomal DNA PCR products derived from proliferating GBM cells. Lanes 2–5 contain samples amplified using NANOG/P8-SmaI-3’-UTR-F2/R2 (Primer set III). Lanes 2 and 4 contain undigested samples (controls) and lanes 3 and 5 contain samples digested with SmaI. As the pCR4-TOPO-TA vector lacks SmaI restriction enzyme sites, the digestion and linearization by SmaI is a confirmation for a positive clone. B. BLAST analysis of the clone of exosomal DNA PCR products derived from proliferating human neural stem cells seen in A. This pCR4-TOPO-TA vector clone contains the PCR fragment produced using NANOG/P8-SmaI-3’-UTR-F2/R2 (Primer set III). SmaI restriction enzyme site is indicated by a box. C. Analysis of the clone of exosomal DNA PCR products derived from proliferating GBM cells seen in A. This pCR4-TOPO-TA vector clone contains the PCR fragment produced using NANOG/P8-SmaI-3’-UTR-F2/R2 (Primer set III). SmaI site is indicated by a box. (PDF) [file pone.0197782.s002.pdf]

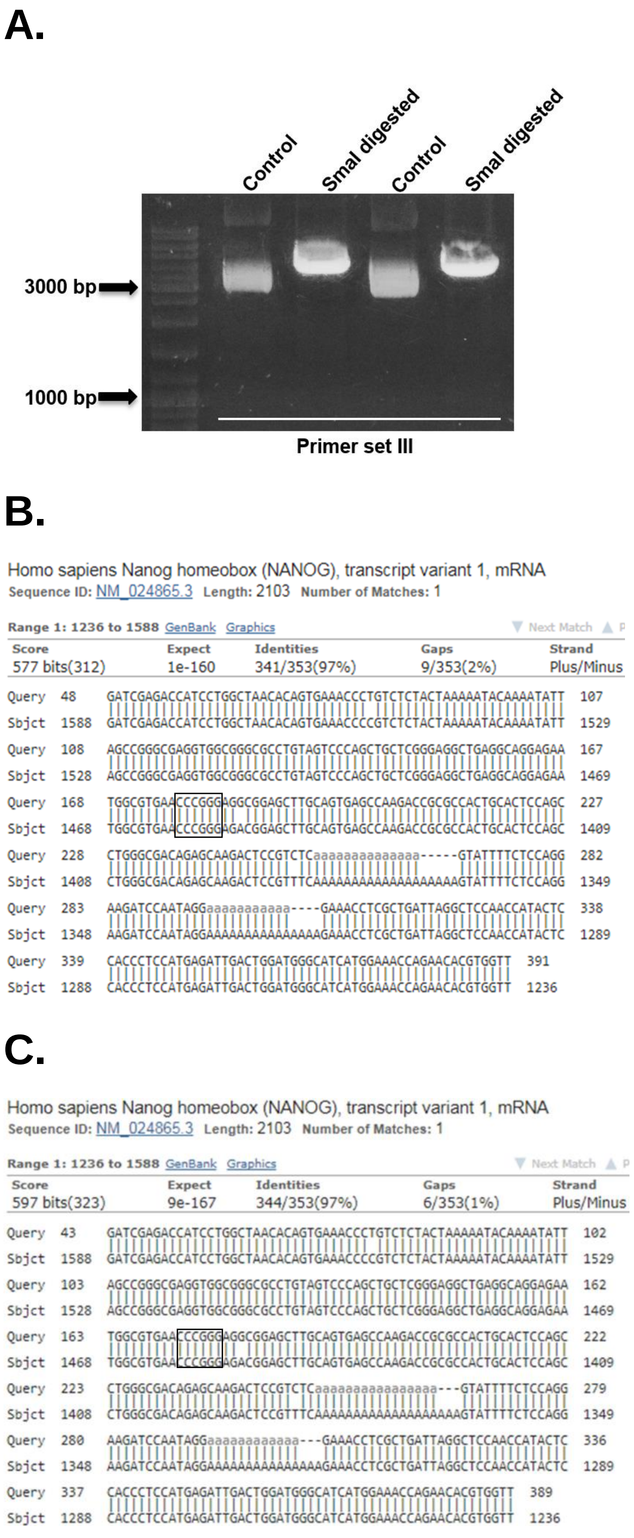
A.
Control
SmaI digested
Control
SmaI digested
3000 bp
1000 bp
Primer set III
B.
Homo sapiens Nanog homeobox (NANOG), transcript variant 1, mRNA
Sequence ID: NM_024865.3 Length: 2103 Number of Matches: 1
Range 1: 1236 to 1588 GenBank Graphics
Next Match
Score 577 bits(312) Expect 1e-160 Identities 341/353(97%) Gaps 9/353(2%) Strand Plus/Minus
Query 48 GATCGAGACCATCCTGGCTAACACAGTGAAACCCTGTCTCTACTAAAAATACAAAATATT 107
Sbjct 1588 GATCGAGACCATCCTGGCTAACACAGTGAAACCCCGTCTCTACTAAAAATACAAAATATT 1529
Query 108 AGCCGGGCGAGGTGGCGGGCGCCTGTAGTCCCAGCTGCTCGGGAGGCTGAGGCAGGAGAA 167
Sbjct 1528 AGCCGGGCGAGGTGGCGGGCGCCTGTAGTCCCAGCTGCTCGGGAGGCTGAGGCAGGAGAA 1469
Query 168 TGGCGTGAACCCGGGAGGCGGAGCTTGCAGTGAGCCAAGACCGCGCCACTGCACTCCAGC 227
Sbjct 1468 TGGCGTGAACCCGGGAGACGGAGCTTGCAGTGAGCCAAGACCGCGCCACTGCACTCCAGC 1409
Query 228 CTGGGCGACAGAGCAAGACTCCGTCTCaaaaaaaaaaaaaa-----GTATTTTCTCCAGG 282
Sbjct 1408 CTGGGCGACAGAGCAAGACTCCGTTTCAAAAAAAAAAAAAAAAAAAGTATTTTCTCCAGG 1349
Query 283 AAGATCCAATAGGaaaaaaaaaaa----GAAACCTCGCTGATTAGGCTCCAACCATACTC 338
Sbjct 1348 AAGATCCAATAGGAAAAAAAAAAAAAAAGAAACCTCGCTGATTAGGCTCCAACCATACTC 1289
Query 339 CACCCTCCATGAGATTGACTGGATGGGCATCATGGAAACCAGAACACGTGGTT 391
Sbjct 1288 CACCCTCCATGAGATTGACTGGATGGGCATCATGGAAACCAGAACACGTGGTT 1236
C.
Homo sapiens Nanog homeobox (NANOG), transcript variant 1, mRNA
Sequence ID: NM_024865.3 Length: 2103 Number of Matches: 1
Range 1: 1236 to 1588 GenBank Graphics
Next Match
Score 597 bits(323) Expect 9e-167 Identities 344/353(97%) Gaps 6/353(1%) Strand Plus/Minus
Query 43 GATCGAGACCATCCTGGCTAACACAGTGAAACCCTGTCTCTACTAAAAATACAAAATATT 102
Sbjct 1588 GATCGAGACCATCCTGGCTAACACAGTGAAACCCCGTCTCTACTAAAAATACAAAATATT 1529
Query 103 AGCCGGGCGAGGTGGCGGGCGCCTGTAGTCCCAGCTGCTCGGGAGGCTGAGGCAGGAGAA 162
Sbjct 1528 AGCCGGGCGAGGTGGCGGGCGCCTGTAGTCCCAGCTGCTCGGGAGGCTGAGGCAGGAGAA 1469
Query 163 TGGCGTGAACCCGGGAGGCGGAGCTTGCAGTGAGCCAAGACCGCGCCACTGCACTCCAGC 222
Sbjct 1468 TGGCGTGAACCCGGGAGACGGAGCTTGCAGTGAGCCAAGACCGCGCCACTGCACTCCAGC 1409
Query 223 CTGGGCGACAGAGCAAGACTCCGTCTCaaaaaaaaaaaaaaaa---GTATTTTCTCCAGG 279
Sbjct 1408 CTGGGCGACAGAGCAAGACTCCGTTTCAAAAAAAAAAAAAAAAAAAGTATTTTCTCCAGG 1349
Query 280 AAGATCCAATAGGaaaaaaaaaaaa---GAAACCTCGCTGATTAGGCTCCAACCATACTC 336
Sbjct 1348 AAGATCCAATAGGAAAAAAAAAAAAAAAGAAACCTCGCTGATTAGGCTCCAACCATACTC 1289
Query 337 CACCCTCCATGAGATTGACTGGATGGGCATCATGGAAACCAGAACACGTGGTT 389
Sbjct 1288 CACCCTCCATGAGATTGACTGGATGGGCATCATGGAAACCAGAACACGTGGTT 1236
