# Supplementary material for: Differential sequences of exosomal NANOG DNA as a potential diagnostic cancer marker
Source: PLoS One. 2018 May 22;13(5):e0197782. doi: 10.1371/journal.pone.0197782 (PMC5963750; doi:10.1371/journal.pone.0197782)
Supplement: S3 Fig — Comparison of PCR product of exosomal DNA derived from small cell lung cancer CRL5903 with ‘NANOG Homo sapiens mRNA for homeobox transcription factor Nanog, complete cds’ (GenBank: AB093576.1). The exosomal DNA was amplified with NANOG/P8-3’UTR-F2/R2 (Primer set IV) and cloned into pCR4-TOPO-TA vector. The PCR product contains a sequence of 22 bp (indicated by a box) not reported in NANOG mRNA variants. This 22bp sequence is reported within NANOGP1 intron from positions 4097–4118 and within NANOGP1 exon from positions 6889–6909. (PDF) [file pone.0197782.s003.pdf]

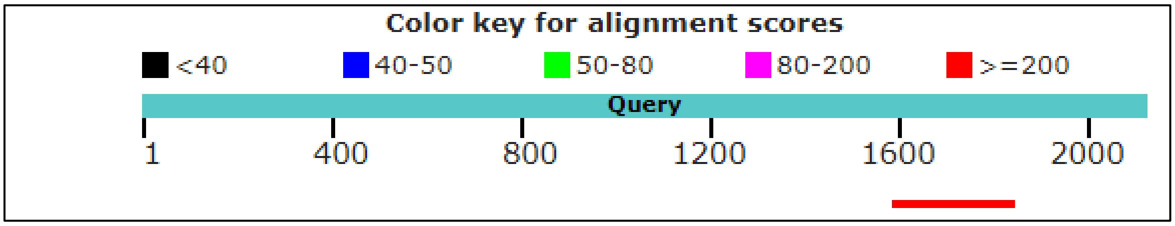

Sequence ID: Query_46487 Length: 308 Number of Matches: 1

Range 1: 47 to 308 Graphics ▼ Next Match ▲ P

Score	Expect	Identities	Gaps	Strand
484 bits(262)	6e-141	262/262(100%)	0/262(0%)	Plus/Minus

```
Query  1576  CGATCTCCTGACCTTGTGATCCGCCCGCCTCGGCCTCCCTAACAGCTGGGATTACAGGCG  1635
             ||||||||||||||||||||||||||||||||||||||||||||||||||||||||||||
Sbjct  308   CGATCTCCTGACCTTGTGATCCGCCCGCCTCGGCCTCCCTAACAGCTGGGATTACAGGCG  249

Query  1636  TGAGCCACCGCGCCCTGCCTAGAAAAGACATTTTAATAACCTTGGCTGCTAAGGACAACA  1695
             ||||||||||||||||||||||||||||||||||||||||||||||||||||||||||||
Sbjct  248   TGAGCCACCGCGCCCTGCCTAGAAAAGACATTTTAATAACCTTGGCTGCTAAGGACAACA  189

Query  1696  TTGATAGAAGCCGTCTCTGGCTATAGATAAGTAGATCTAATACTAGTTTGGATATCTTTA  1755
             ||||||||||||||||||||||||||||||||||||||||||||||||||||||||||||
Sbjct  188   TTGATAGAAGCCGTCTCTGGCTATAGATAAGTAGATCTAATACTAGTTTGGATATCTTTA  129

Query  1756  GGGTTTAGAATCTAACCTCAAGAATAAGAAATACAAGTACGAATTGGTGATGAAGATGTA  1815
             ||||||||||||||||||||||||||||||||||||||||||||||||||||||||||||
Sbjct  128   GGGTTTAGAATCTAACCTCAAGAATAAGAAATACAAGTACGAATTGGTGATGAAGATGTA  69

Query  1816  TTCGTATTGTTTGGGATTGGGA  1837
             ||||||||||||||||||||||
Sbjct  68    TTCGTATTGTTTGGGATTGGGA  47
```
