# Supplementary material for: Differential sequences of exosomal NANOG DNA as a potential diagnostic cancer marker
Source: PLoS One. 2018 May 22;13(5):e0197782. doi: 10.1371/journal.pone.0197782 (PMC5963750; doi:10.1371/journal.pone.0197782)
Supplement: S4 Fig — Comparison of PCR product of exosomal DNA derived from small cell lung cancer CRL5903 with ‘NANOG genome sequence Homo sapiens chromosome 12, GRCh38.p7’ (NCBI Reference Sequence: NC_000012.12). The exosomal DNA was amplified with NANOG/P8-3’UTR-F2/R2 (Primer set IV) and cloned into pCR4-TOPO-TA vector. The PCR product contains a sequence of 22 bp (indicated by a box) not reported in NANOG genomic DNA. This 22bp sequence is reported within NANOGP1 intron from positions 4097–4118 and within NANOGP1 exon from positions 6889–6909. (PDF) [file pone.0197782.s004.pdf]

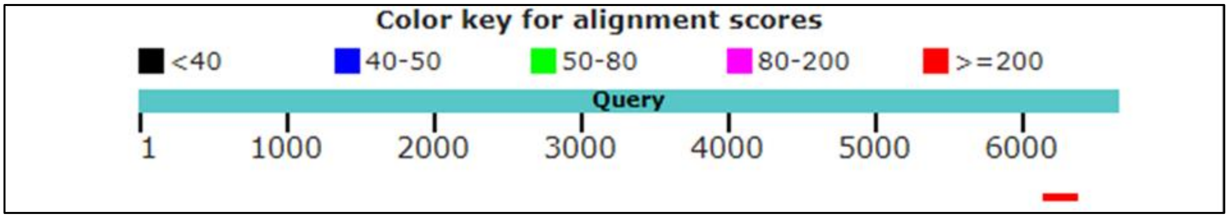

Sequence ID: Query_82977 Length: 308 Number of Matches: 1

Range 1: 47 to 308 Graphics ▼ Next Match ▲ Pre

Score	Expect	Identities	Gaps	Strand
326 bits(176)	1e-92	238/263(90%)	23/263(8%)	Plus/Minus

```
Query  6147  CGATCTCCTGACCTTGTGATCCACCCGCCTCGGCCTCCCTAACAGCTGGGATTTACAGGC  6206
             |||||||||||||||||||||| |||||||||||||||||||||||||||||| ||||||
Sbjct  308   CGATCTCCTGACCTTGTGATCCGCCCGCCTCGGCCTCCCTAACAGCTGGGATT-ACAGGC  250

Query  6207  GTGAGCCACCGCGCCCTGCCTAGAAAAGACATTTTAATAACCTTGGCTGC----------  6256
             ||||||||||||||||||||||||||||||||||||||||||||||||||
Sbjct  249   GTGAGCCACCGCGCCCTGCCTAGAAAAGACATTTTAATAACCTTGGCTGCTAAGGACAAC  190

Query  6257  ------------CGTCTCTGGCTATAGATAAGTAGATCTAATACTAGTTTGGATATCTTT  6304
                         ||||||||||||||||||||||||||||||||||||||||||||||||
Sbjct  189   ATTGATAGAAGCCGTCTCTGGCTATAGATAAGTAGATCTAATACTAGTTTGGATATCTTT  130

Query  6305  AGGGTTTAGAATCTAACCTCAAGAATAAGAAATACAAGTACAAATTGGTGATGAAGATGT  6364
             ||||||||||||||||||||||||||||||||||||||||| ||||||||||||||||||
Sbjct  129   AGGGTTTAGAATCTAACCTCAAGAATAAGAAATACAAGTACGAATTGGTGATGAAGATGT  70

Query  6365  ATTCGTATTGTTTGGGATTGGGA  6387
             |||||||||||||||||||||||
Sbjct  69    ATTCGTATTGTTTGGGATTGGGA  47
```
