# Supplementary material for: Differential sequences of exosomal NANOG DNA as a potential diagnostic cancer marker
Source: PLoS One. 2018 May 22;13(5):e0197782. doi: 10.1371/journal.pone.0197782 (PMC5963750; doi:10.1371/journal.pone.0197782)
Supplement: S5 Fig — Comparison of PCR product of exosomal DNA derived from small cell lung cancer CRL5903 with A. ‘Homo sapiens Nanog homeobox (NANOG), transcript variant 1, mRNA’ (NCBI Reference Sequence: NM_024865) and B. ‘Homo sapiens Nanog homeobox (NANOG), transcript variant 2, mRNA’ (NCBI Reference Sequence: NM_001297698.1). The exosomal DNA was amplified with NANOG/P8-3’UTR-F2/R2 (Primer set IV) and cloned into pCR4-TOPO-TA vector. The PCR products contain a sequence of 22 bp (indicated by a box) not reported in NANOG mRNA transcript variant 1 and 2. This 22bp sequence is reported within NANOGP1 intron from positions 4097–4118 and within NANOGP1 exon from positions 6889–6909. (PDF) [file pone.0197782.s005.pdf]

A.

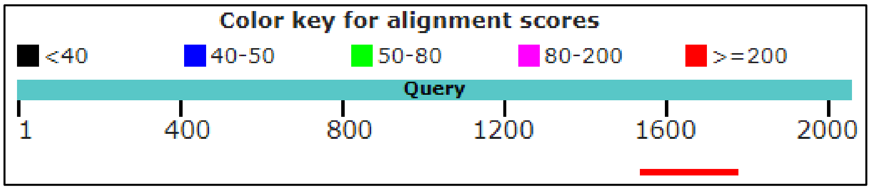


```
Sequence ID: Query_190907  Length: 308  Number of Matches: 1

Range 1: 47 to 308 Graphics                                  Next Match  Pr

Score            Expect   Identities       Gaps          Strand
326 bits(176)    4e-93    238/263(90%)     23/263(8%)    Plus/Minus

Query  1584  CGATCTCCTGACCTTGTGATCCACCCGCCTCGGCCTCCCTAACAGCTGGGATTTACAGGC  1643
             |||||||||||||||||||||| ||||||||||||||||||||||||||||||| ||||||
Sbjct  308   CGATCTCCTGACCTTGTGATCCGCCCGCCTCGGCCTCCCTAACAGCTGGGATT-ACAGGC  250

Query  1644  GTGAGCCACCGCGCCCTGCCTAGAAAAGACATTTTAATAACCTTGGCTGC----------  1693
             ||||||||||||||||||||||||||||||||||||||||||||||||||
Sbjct  249   GTGAGCCACCGCGCCCTGCCTAGAAAAGACATTTTAATAACCTTGGCTGCTAAGGACAAC  190

Query  1694  ------------CGTCTCTGGCTATAGATAAGTAGATCTAATACTAGTTTGGATATCTTT  1741
                         ||||||||||||||||||||||||||||||||||||||||||||||||
Sbjct  189   ATTGATAGAAGCCGTCTCTGGCTATAGATAAGTAGATCTAATACTAGTTTGGATATCTTT  130

Query  1742  AGGGTTTAGAATCTAACCTCAAGAATAAGAAATACAAGTACAAATTGGTGATGAAGATGT  1801
             ||||||||||||||||||||||||||||||||||||||||| ||||||||||||||||||
Sbjct  129   AGGGTTTAGAATCTAACCTCAAGAATAAGAAATACAAGTACGAATTGGTGATGAAGATGT  70

Query  1802  ATTCGTATTGTTTGGGATTGGGA  1824
             |||||||||||||||||||||||
Sbjct  69    ATTCGTATTGTTTGGGATTGGGA  47
```

B.

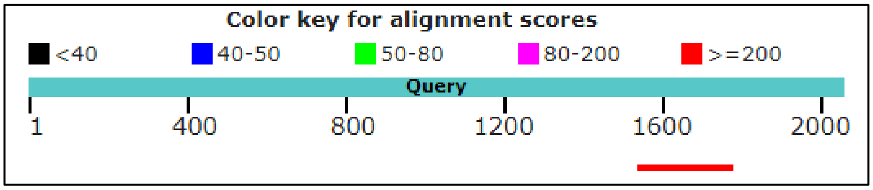


```
Sequence ID: Query_28043  Length: 308  Number of Matches: 1

Range 1: 47 to 308 Graphics                                  Next Match  P

Score            Expect   Identities       Gaps          Strand
326 bits(176)    4e-93    238/263(90%)     23/263(8%)    Plus/Minus

Query  1536  CGATCTCCTGACCTTGTGATCCACCCGCCTCGGCCTCCCTAACAGCTGGGATTTACAGGC  1595
             |||||||||||||||||||||| ||||||||||||||||||||||||||||||| ||||||
Sbjct  308   CGATCTCCTGACCTTGTGATCCGCCCGCCTCGGCCTCCCTAACAGCTGGGATT-ACAGGC  250

Query  1596  GTGAGCCACCGCGCCCTGCCTAGAAAAGACATTTTAATAACCTTGGCTGC----------  1645
             ||||||||||||||||||||||||||||||||||||||||||||||||||
Sbjct  249   GTGAGCCACCGCGCCCTGCCTAGAAAAGACATTTTAATAACCTTGGCTGCTAAGGACAAC  190

Query  1646  ------------CGTCTCTGGCTATAGATAAGTAGATCTAATACTAGTTTGGATATCTTT  1693
                         ||||||||||||||||||||||||||||||||||||||||||||||||
Sbjct  189   ATTGATAGAAGCCGTCTCTGGCTATAGATAAGTAGATCTAATACTAGTTTGGATATCTTT  130

Query  1694  AGGGTTTAGAATCTAACCTCAAGAATAAGAAATACAAGTACAAATTGGTGATGAAGATGT  1753
             ||||||||||||||||||||||||||||||||||||||||| ||||||||||||||||||
Sbjct  129   AGGGTTTAGAATCTAACCTCAAGAATAAGAAATACAAGTACGAATTGGTGATGAAGATGT  70

Query  1754  ATTCGTATTGTTTGGGATTGGGA  1776
             |||||||||||||||||||||||
Sbjct  69    ATTCGTATTGTTTGGGATTGGGA  47
```
