# Supplementary material for: Differential sequences of exosomal NANOG DNA as a potential diagnostic cancer marker
Source: PLoS One. 2018 May 22;13(5):e0197782. doi: 10.1371/journal.pone.0197782 (PMC5963750; doi:10.1371/journal.pone.0197782)
Supplement: S6 Fig — Comparison of PCR product of exosomal DNA derived from small cell lung cancer CRL5903 with ‘NANOGP8 cds Homo sapiens isolate NA07038*D2-1 NANOGP8 (NANOGP8) gene, complete cds’ (GenBank: JX104846.1). The exosomal DNA was amplified with NANOG/P8-3’UTR-F2/R2 (Primer set IV) and cloned into pCR4-TOPO-TA vector. The PCR product and NANOGP8 cds share 100 base pairs of homology. (PDF) [file pone.0197782.s006.pdf]

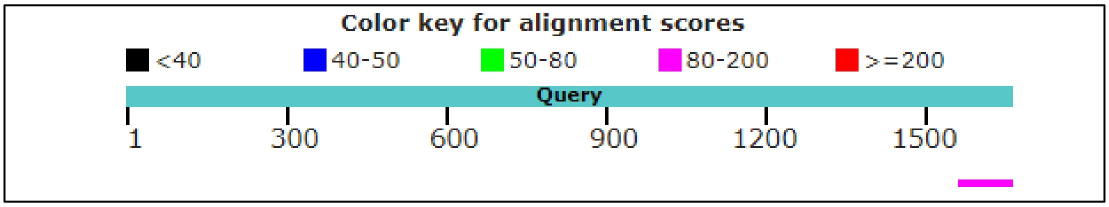

Sequence ID: Query_221727 Length: 308 Number of Matches: 1

Range 1: 208 to 308 Graphics Next Match Pr

Score	Expect	Identities	Gaps	Strand
182 bits(98)	7e-50	101/102(99%)	1/102(0%)	Plus/Minus

```
Query  1550  CGATCTCCTGACCTTGTGATCCGCCCGCCTCGGCCTCCCTAACAGCTGGGATTTACAGGC  1609
             ||||||||||||||||||||||||||||||||||||||||||||||||||||| ||||||
Sbjct  308   CGATCTCCTGACCTTGTGATCCGCCCGCCTCGGCCTCCCTAACAGCTGGGATT-ACAGGC  250

Query  1610  GTGAGCCACCGCGCCCTGCCTAGAAAAGACATTTTAATAACC  1651
             ||||||||||||||||||||||||||||||||||||||||||
Sbjct  249   GTGAGCCACCGCGCCCTGCCTAGAAAAGACATTTTAATAACC  208
```
