# Supplementary material for: Differential sequences of exosomal NANOG DNA as a potential diagnostic cancer marker
Source: PLoS One. 2018 May 22;13(5):e0197782. doi: 10.1371/journal.pone.0197782 (PMC5963750; doi:10.1371/journal.pone.0197782)
Supplement: S7 Fig — Comparison of PCR product of exosomal DNA derived from small cell lung cancer CRL5903 with ‘Homo sapiens Nanog homeobox pseudogene 8 (NANOGP8) on chromosome 15’ (NCBI Reference Sequence: NG_004093.3). The exosomal DNA was amplified with NANOG/P8-3’UTR-F2/R2 (Primer set IV) and cloned into pCR4-TOPO-TA vector. The PCR product contains a sequence of 22 bp (indicated by a box) not reported in genomic NANOGP8. This 22bp sequence is reported within NANOGP1 intron from positions 4097–4118 and within NANOGP1 exon from positions 6889–6909. (PDF) [file pone.0197782.s007.pdf]

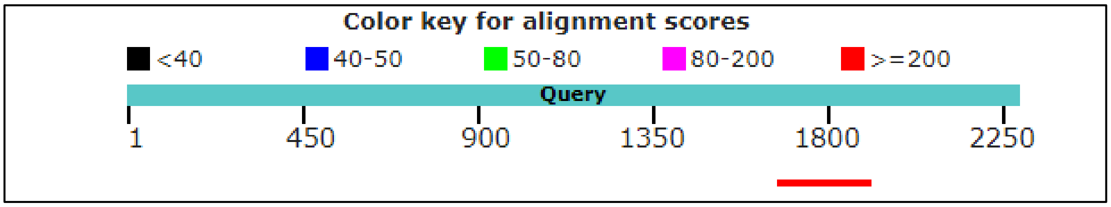

Sequence ID: Query_49117 Length: 308 Number of Matches: 1

Range 1: 47 to 308 Graphics

Next Match Pr

Score	Expect	Identities	Gaps	Strand
326 bits(176)	4e-93	238/263(90%)	23/263(8%)	Plus/Minus

```
Query  1666  CGATCTCCTGACCTTGTGATCCGCCCGCCTCGGCCTCCCTAACAGCTGGGATTTACAGGC  1725
             ||||||||||||||||||||||||||||||||||||||||||||||||||||| ||||||
Sbjct  308   CGATCTCCTGACCTTGTGATCCGCCCGCCTCGGCCTCCCTAACAGCTGGGATT-ACAGGC  250

Query  1726  GTGAGCCACCGCGCCCTGCCTAGAAAAGACATTTTAATAACCTTGGCTGC----------  1775
             ||||||||||||||||||||||||||||||||||||||||||||||||||          
Sbjct  249   GTGAGCCACCGCGCCCTGCCTAGAAAAGACATTTTAATAACCTTGGCTGCTAAGGACAAC  190

Query  1776  ------------CGTCTCTGGCTATAGATAAGTAGATCTAATACGAGTTTGGATATCTTT  1823
                         |||||||||||||||||||||||||||||||| |||||||||||||||
Sbjct  189   ATTGATAGAAGCCGTCTCTGGCTATAGATAAGTAGATCTAATACTAGTTTGGATATCTTT  130

Query  1824  AGGGTTTAGAATCTAACCTCAAGAATAAGAAATACAAGTACAAATTGGTGATGAAGATGT  1883
             ||||||||||||||||||||||||||||||||||||||||| ||||||||||||||||||
Sbjct  129   AGGGTTTAGAATCTAACCTCAAGAATAAGAAATACAAGTACGAATTGGTGATGAAGATGT  70

Query  1884  ATTCGTATTGTTTGGGATTGGGA  1906
             |||||||||||||||||||||||
Sbjct  69    ATTCGTATTGTTTGGGATTGGGA  47
```
